# Marker-Based Structural Displacement Measurement Models with Camera Movement Error Correction Using Image Matching and Anomaly Detection

**DOI:** 10.3390/s20195676

**Published:** 2020-10-05

**Authors:** Jisung Kim, Youngdo Jeong, Hyojin Lee, Hongsik Yun

**Affiliations:** 1 School of Civil, Architectural Engineering & Landscape Architecture, Sungkyunkwan University, Suwon 16419, Korea; jskim6687@skku.edu; 2 Infra Research Group, R&D Center, POSCO E&C, Incheon 21985, Korea; jyd@poscoenc.com; 3 Institute of Geographic & Environmental Technology, GEOMEXSOFT Ltd., Chuncheon 24461, Korea; tallwind@geomex.co.kr

**Keywords:** camera movement error, image matching, anomaly detection, marker-based displacement measurement, error correction

## Abstract

To prevent collapse accidents at construction sites, the marker-based displacement measurement method was developed. However, it has difficulty in obtaining accurate measurements at long distances (>50 m) in an outdoor environment because of camera movements. To overcome this problem, marker-based structural displacement measurement models using image matching and anomaly detection were designed in this study. Then, the performance of each model in terms of camera movement error correction was verified through comparison with that of a conventional model. The results show that the systematic errors due to camera movements (<1.7°) were corrected. The detection rate of markers with displacement reached 95%, and the probability that the error size would be less than 10 mm was ≥ 95% with a 95% confidence interval at a distance of more than 100 m. Moreover, the normalized mean square error was less than 0.1. The models developed in this study can measure the pure displacement of an object without the systematic errors caused by camera movements. Furthermore, these models can be used to measure the displacements of distant structures using closed-circuit television cameras and markers in an outdoor environment with high accuracy.

## 1. Introduction

Collapse accidents at construction sites are continuously occurring [1,2,3]. According to Heinrich’s law, a small local displacement occurs in a structure before a large-scale collapse accident occurs [4,5,6]. Detecting such small local displacements can assist in effectively preventing large-scale collapse accidents. For this reason, various techniques have been studied to measure the displacement of structures, such as using sensors inside structures, e.g., displacement gauges and clinometers [7,8,9,10,11,12], for short-period displacements, and using laser scanners [13,14,15,16], total stations [17], the Global Navigation Satellite System (GNSS) [17,18,19], and images [15,19,20,21,22,23,24,25,26,27] for long-period displacements.

Methods using images can measure the displacement of a structure by identifying the movement of a feature point of a structure from images obtained using a camera [26,27]. Owing to the advent of digital cameras and their high reliability, the development of highly accurate computer image processing technology and displacement measurement systems using images is being researched. Displacement measurement systems have the advantages of being low cost and fast; moreover, they require simple procedures and generate accurate outputs [21,23,26,27].

Displacement measurement using images mainly involves extracting feature points and matching the extracted points [19] between the initial and final images. However, incorrect matching of feature points may occur when it is performed automatically. To prevent such point matching errors, methods of attaching artificial markers have been proposed [24,25,26,27]. The marker-based displacement measurement method uses a marker, as opposed to the marker-less approach. In this method, markers are extracted as feature points, and since each marker has its own value [28], the error due to feature point extraction can be reduced.

Research on the marker-based displacement measurement method has been steadily increasing since the advent of aerial photogrammetry. Cho and Sim [29] attached a marker to a pier and determined its deformation by measuring the marker using photographs. At that time, because a film camera was used instead of a digital camera, there were some limitations. Hwang [24] presented a measurement model that combines several measurement methods, such as GNSS and marker-based displacement measurement, to measure the displacement of a structure and applied the model to short-period displacements of structures. In a study by Cho et al. [30], marker-based displacement measurements were performed at an actual construction site. However, since both Hwang [24] and Cho et al. [30] had not conducted experiments at long distances (>100 m), it is difficult to determine the usability of their methods at long distances. Luis et al. [31] performed marker-based displacement measurements with an accuracy of less than 30 mm at a distance of ≥500 m using a telephoto lens and an LED light-emitting marker. In their process, a solid beam (stiffening beam) was installed to fix the camera tightly to prevent camera movement. Subsequently, Cong et al. [25] and Suhovilov et al. [26] studied a marker-based structural displacement measurement method and conducted studies at a short distance (<50 m) to exclude camera movement errors.

As can be observed from the aforementioned studies, some errors were evident, such as marker recognition errors due to illumination and environmental factors [25,26,27] and errors due to camera movement [25,26,27,32], because the marker-based displacement measurement method uses optical images. For this reason, this method is not suitable for long-duration and long-distance measurements [21,22,25,26,27].

To overcome the above limitation, techniques for correcting recognition reduction in markers and feature points are necessary. Currently, owing to improvements in computer performance and the development of machine learning technology, such techniques have increasingly progressed [28,33,34]. Moreover, methods for camera movement error correction are also constantly being studied in view of the development of image processing technology and machine learning [23,25,32,35,36,37,38]. In particular, image stabilization and image matching can compensate for errors due to camera movement [36,37,38]. Satoru and Hiroki [35] analyzed the correlation between camera movement and bridge displacement error and calibrated the camera based on this correlation. The camera was installed at a distance of 7–9 m from the bridge being observed and panning was performed at 0.6°. The observation error was within 2 mm. Asal [36] studied video stabilization using a point feature matching technology and corrected the error caused by camera movement. Cong et al. [25] corrected errors related to camera movement by using two cameras instead of one. The camera’s motion angle ranged from 0 to 1.2°, and the experiment was conducted in an environment where the distance between the object and the camera was within 1 m. In a study by Ali and David [37], the distance between the camera and the object to be observed was not specified, but camera motion errors were corrected using image stabilization and optical flow. Ekkaphon and Weerawat [38] implemented image stabilization of drone cameras by extracting feature points using speeded-up robust features (SURF) and calculating the transformation vector of feature points. Jeong et al. [32] proposed an error correction method using a grid. Shichao et al. [23] corrected the image motion error due to temperature by decomposing it into vectors. Recently, Qui et al. [39] installed an inertial measurement unit (IMU) on a camera to infer the camera movement and performed a study on correcting the camera error.

As observed from previous studies, long-distance measurement at an actual construction site is not feasible unless the systematic errors due to camera movements are eliminated. Therefore, to apply a marker-based displacement measurement system to an actual construction site, it is necessary to eliminate such errors. Thus, this study aims to develop marker-based measurement models using image matching and anomaly detection with camera movement error correction.

The models proposed in this study mainly compensate for camera movements during measurement. They are designed for correcting the camera movement and calculating the marker displacement using two images obtained from only one camera as well as two or more cameras, because they do not need stereography. Using only one camera to measure distance makes installation cost lower and enhance its usability. Consequently, this study has the following limitations:

- It is assumed that the structural displacement occurs on a two-dimensional plane, because a marker-based measurement system is not suitable for 3D displacement with high accuracy (less than a few centimeters) in a long distance (>50 m).
- The displacement occurs in anomaly.
- The structure does not experience rigid motion.
- Camera movements due to the external environment mainly occur in the left and right (panning) and up and down (tilting) directions, but rotation, focus change, and zoom level change are not considered.

The purpose of this study was to develop displacement measurement models using image matching and anomaly detection for correcting the systematic errors caused by camera movements. To achieve this purpose, three models were designed:

- Model 1: Using image matching.
- Model 2: Using anomaly detection.
- Model 3: Using both image matching and anomaly detection.

**Figure 1:**
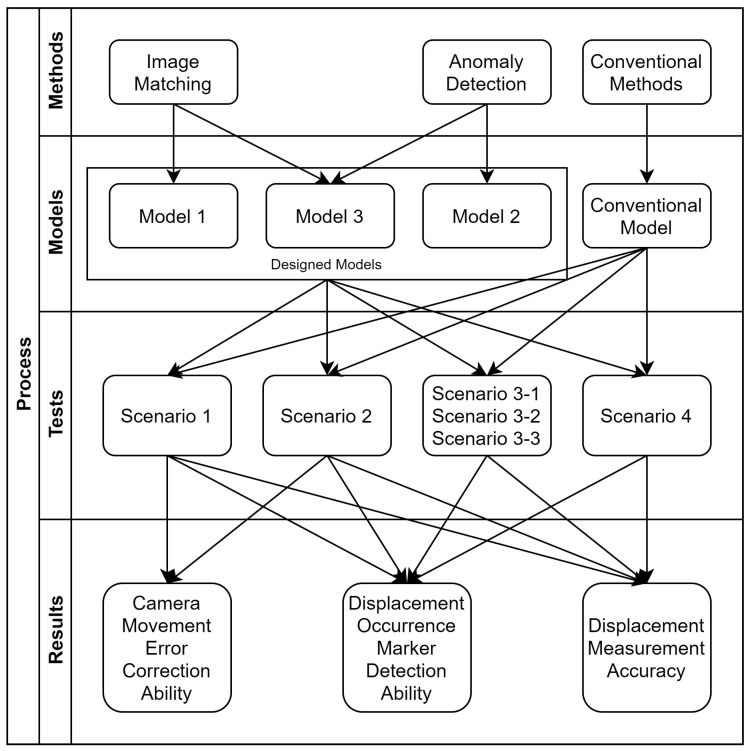
Research methodology.

To verify the performance of each model, six experimental scenarios with different procedures and conditions for each scenario were designed (Figure 1). In this study, the following indices were used to evaluate the performance of the models:

1. Camera movement error correction performance using fixed markers.
2. Detection of markers with displacement.
3. Displacement measurement accuracy of the moved markers when camera movements occur.

The structure of this article is as follows: Section 2 describes the procedures for developing the marker-based displacement measurement models and for verifying their performance. Section 3 and Section 4 presents the results and discussion, respectively. Finally, Section 5 summarizes the conclusions of the study.

## 2. Materials and Methods

### 2.1. Preliminary Works and Methodology

#### 2.1.1. Marker-Based Displacement Measurement

**Figure 2:**
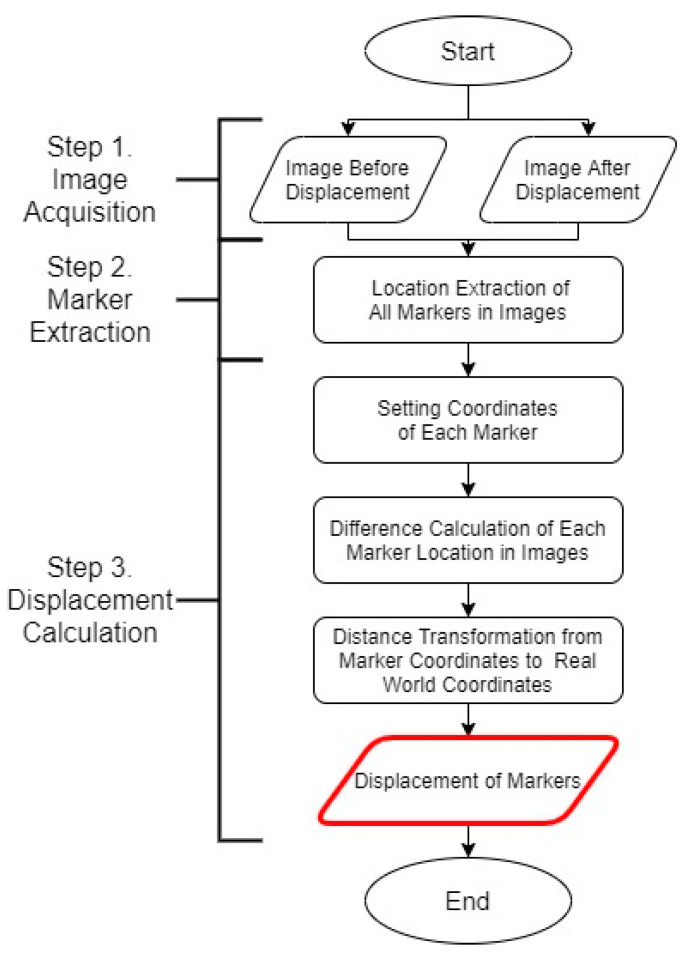
Conventional model of marker-based displacement measurement.

As mentioned in the introduction, marker-based displacement measurement involves attaching markers to a structure and obtaining images of the markers with a camera. Then, using the images with markers, the structural displacements are calculated. The conventional model [24,26,33] of the marker-based displacement measurement can be divided into three steps (Figure 2):

1. Image acquisition step;
2. Marker extraction step;
3. Displacement calculation step.

Among them, the step in which errors occur due to camera movements in the conventional model is step 1. In the image acquisition step, initial and final images of a specific section are acquired. The initial image of the section is the reference, whereas the final one represents the image of the specific time at which the displacement is to be measured. The model calculates only the displacement that occurs within the section. In the case of using a single camera, the measurement is considered accurate when all attributes, such as zoom level, number of pixels, image range, principal point location, and camera direction, of the initial and final images match. Camera movement errors occur during this image acquisition step. These errors impede matching of the attributes between the initial and final images, resulting in an inaccurate displacement measurement result.

In the marker extraction step, the outlines of the markers in the images are detected and their positions are extracted. The markers used for measurement contain information on marker patterns, such as bar codes and QR codes. This information can reduce both the marker recognition error and the feature point extraction error. Additionally, it can provide additional data necessary for measurement. The pattern of markers used in this study is “ARUco,” which was developed by Garrido-Jurado et al. [40]. This pattern has the advantage of easy marker generation and high recognition rate even in an environment that causes occlusion.

**Figure 3:**
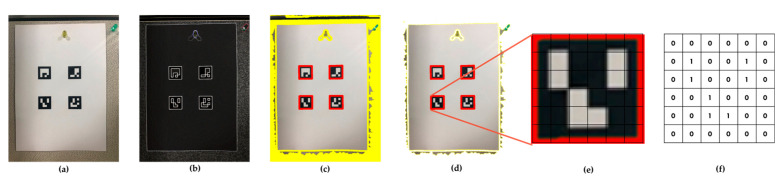
Process for automatic marker detection in an image: (**a**) original image; (**b**) result of applying local thresholding; (**c**) contour detection; (**d**) polygonal approximation and removal of irrelevant contours; (**e**) example of marker after perspective transformation; (**f**) bit assignment for each cell. [40].

The process of extracting markers in an image is shown in Figure 3. First, the image of the RGB channel (Figure 3a) is converted into a grayscale image of a single channel. Then, adaptive thresholding is used to extract the shape boundary from a single-channel grayscale image (Figure 3b). By creating a contour using Satoshi’s algorithm [41], objects representing distinct boundaries can be extracted. Among the objects detected through the contour, there are objects that are not markers. Thus, only rectangular contour objects with four vertices are detected by the Douglas–Peuker method [42]. The detected rectangular contour objects are transformed into a square shape, as depicted in Figure 3e, using a homographic matrix. The 36 cells constituting the square ARUco marker must have a value of 0 or 1, but each square obtained from an image has a bimodal distribution with a mode of 0 and 1. Therefore, Otsu’s method [43] is applied so that each element will have a value of 0 or 1, and a binary array (as displayed in Figure 3f) is obtained. By reading the binary array, the marker information can be obtained.

Although the relative position and posture between the camera and the marker can be estimated through the position and information of the marker in the image, it is difficult to calculate the exact relative position due to errors caused by the camera *z*-axis over a long distance. Therefore, achieving an accurate displacement measurement will be a problem. To solve this, the following method is used.

**Figure 4:**
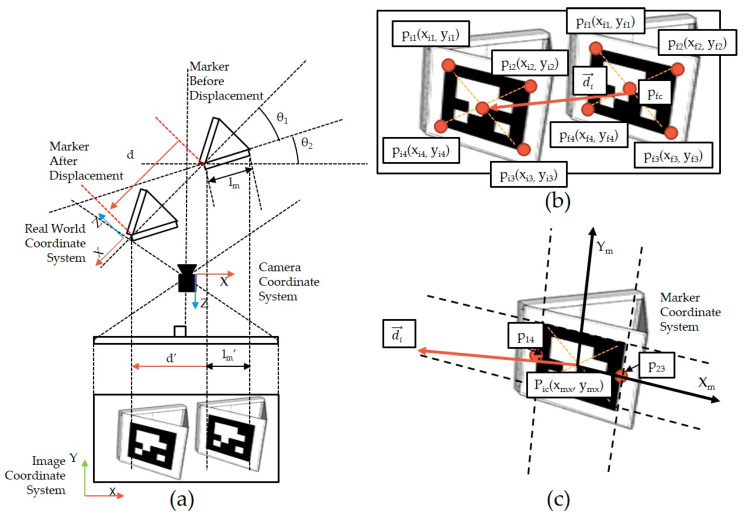
Marker displacement measurement image: (**a**) arrangement of marker, camera, and image during the displacement event; (**b**) coordinates of marker vertices in the image coordinate system before and after the displacement event; (**c**) marker coordinate system in the image.

In the real world, it is assumed that the relationships between the marker, camera, and image before and after displacement are as illustrated in Figure 4a. The positions of the marker vertices in the initial and final images can be obtained during the marker extraction step. The vertex coordinates $p_{i}$ in the initial image of the marker before the displacement occur and the vertex coordinates $p_{f}$ in the final image after occurrence of the displacement are indicated in Figure 4b.

Because the marker face is two-dimensional, a two-dimensional plane Cartesian coordinate system can be used on the marker plane, and this is called the marker coordinate system, which is depicted in Figure 4c.

When displacement d occurs in the real world (Figure 4a), it is denoted as $\vec{d_{i}}$ in the image; $\vec{d_{i}}$ can be divided into an *x*-axis vector $\vec{X_{m}}$ and an *y*-axis vector $\vec{Y_{m}}$ (Figure 4c). In this study, when the camera and marker are installed, the angle is adjusted so that $\vec{d_{i}}$ is parallel to $\vec{X_{m}}$. This setting must be considered when designing the direction of marker attachment according to the direction of structural displacement.

Let $p_{i1}$, $p_{i2}$,$\;p_{i3}$, and $p_{i4}$ be four vertexes that are the left-top, right-top, right-bottom, and left-bottom of the marker before moved, respectively. Furthermore, $p_{14}$ is the center of $p_{i1}$ and $p_{i4}$, and $p_{23}$ is the center of $p_{i2}$ and $p_{i3}$.

Because the marker has a square shape and the marker coordinate system is set according to the face of the marker, the vector $\vec{X_{w}}$ representing the width of the marker is parallel to $\vec{X_{m}}$. From the equation of a straight line including the vector $\vec{X_{m}}$, $p_{14}\left(x_{14},\;y_{14}\right)$ and $p_{23}\left(x_{23},\;y_{23}\right)$ can be found, and through this, $\left\|\vec{X_{w}}\right\|$ is derived (Equation (1)).

(1) $$\|\vec{X_{w}}\|=\|\bar{p_{14}p_{23}}\|=\sqrt{{\left(x_{14}-x_{23}\right)}^{2}+{\left(y_{14}-y_{23}\right)}^{2}}$$

Since $\vec{d_{i}}∥\vec{X_{m}}$ and $\vec{X_{w}}∥\vec{X_{m}}$, $\vec{X_{w}}∥\vec{d_{i}}$. Equation (2) is introduced, and the constant k can be calculated using Equation (3).

(2) $$\vec{d_{i}}=k\vec{X_{w}}$$

(3) $$k=\frac{∥\vec{d_{i}}∥}{∥\vec{X_{w}}∥}$$

At a distance of ≥100 m, the difference between the actual length per pixel at the initial position and the final position of the marker is negligible. Let $\theta_{1}$ be the angle between displacement and surface of the marker, and $\theta_{2}$ be the angle between the marker surface and the camera film, such as a charge-coupled device (CCD) or a complementary metal-oxide-semiconductor (CMOS). The actual displacement d can be calculated using the length per pixel in direction $\vec{X_{w}}$, which is calculated using the actual length of the marker $l_{m}$ (Equation (4)).

(4) $$d=l_{m}\frac{∥\vec{d_{i}}∥}{∥\vec{X_{w}}∥}\frac{\cos\theta_{1}}{\cos\left(\theta_{1}+\theta_{2}\right)}=kl_{m}\frac{\cos\theta_{1}}{\cos\left(\theta_{1}+\theta_{2}\right)}$$

Equation (4) is used to calculate the displacement of the marker when there is no camera movement. When camera movement occurs, error $\vec{e}$ due to the movement affects the observation. Let the displacement vector in the image without camera movement be $\vec{d_{i}}$, the error vector due to the camera movement be $\vec{e}$, and the displacement vector measured in the image with the error be $\vec{d_{i}}$′; then, their relationship is given by Equation (5).

(5) $$\vec{d_{i}{}^{\prime }}=\vec{d_{i}}+\vec{e}$$

To determine the cause of error $\vec{e}$, it is necessary to understand the deformation of the coordinate system due to camera movement. The camera movement deforms both the coordinate systems of the camera and image. The left and right movements of the camera (pan) and the vertical movement (tilt) refer to the translation of the image coordinate system. Zoom denotes the change in focal length (scale), and rotation of the *z*-axis of the camera refers to the rotation of the image coordinate system. T is a 3 × 3 translation matrix because of pan ($t_{x}$) and tilt ($t_{y})$, R is a 3 × 3 rotation matrix because of roll (θ), and S is a 3 × 3 scale matrix because of zoom ($s_{x}$ and $s_{y}$). If $p_{ic}\left(x_{ic},y_{ic}\right)$ and $p_{fc}\left(x_{fc},y_{fc}\right)$ are respectively the center points of the marker in the initial and final images (Figure 4b), then their relationship can be expressed as Equation (6) using the translation (T), rotation (R), and size (S) transformation matrices.

(6) $$\begin{array}{cc} p_{fc} & =T\cdot R\cdot S\cdot p_{ic}\\ & =\left[\begin{array}{c} \begin{array}{ccc} 1 & 0 & t_{x}\\ 0 & 1 & t_{y} \end{array}\\ \begin{array}{ccc} 0 & 0 & 1 \end{array} \end{array}\right]\left[\begin{array}{c} \begin{array}{ccc} \cos\theta & -\sin\theta & 0\\ \sin\theta & \cos\theta & 0 \end{array}\\ \begin{array}{ccc} \;0 & \;\;\;\;\;0\;\; & 1 \end{array} \end{array}\right]\left[\begin{array}{c} \begin{array}{ccc} s_{x} & 0 & 0\\ 0 & s_{y} & 0 \end{array}\\ \begin{array}{ccc} 0 & \;0 & 1 \end{array} \end{array}\right]\left[\begin{array}{c} x_{ic}\\ y_{ic}\\ 1 \end{array}\right]\\ & =\left[\begin{array}{c} \begin{array}{ccc} \cos\theta & -\sin\theta & t_{x}\\ \sin\theta & \cos\theta & t_{y} \end{array}\\ \begin{array}{ccc} \;0 & \;\;\;\;\;0\;\; & 1 \end{array} \end{array}\right]\left[\begin{array}{c} \begin{array}{ccc} s_{x} & 0 & 0\\ 0 & s_{y} & 0 \end{array}\\ \begin{array}{ccc} 0 & \;0 & 1 \end{array} \end{array}\right]\left[\begin{array}{c} x_{ic}\\ y_{ic}\\ 1 \end{array}\right] \end{array}$$

Let t be a displacement matrix with two rows and one column, and it includes error $e_{t}$. On the other hand, t is the displacement matrix that we want without the error. By obtaining the transformation matrix (6) due to the camera movement, the error vector can be obtained, as shown in Equations (7) and (8). To obtain the error vector $e_{t}$, image matching and anomaly detection are used in this study.

(7) $$t^{\prime }=t+e_{t}=\left[\begin{array}{c} t_{x}+e_{x}\\ t_{y}+e_{y} \end{array}\right]$$

(8) $$\vec{e}=e_{x}\hat{i}+e_{y}\hat{j}$$

#### 2.1.2. Image Matching

**Figure 5:**
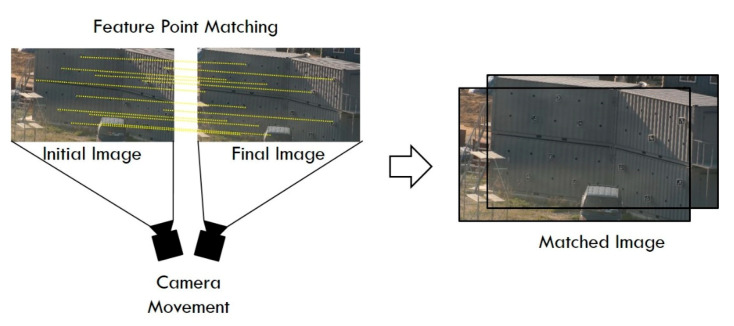
Image matching to correct camera movement error.

Image matching derives the geometric relation between two images by extracting feature points common to the two images (Figure 5). It is used for image stabilization and panoramic image production. The first step in image matching is to extract and match feature points within the images. These feature points should have a clear distinction compared to the surrounding points and have a clear boundary. There are various methods for extracting feature points from images, e.g., SURF, scale invariant feature transform (SIFT), binary robust invariant scalable keypoints (BRISK), maximally stable extremal regions (MSER), Harris, features from accelerated segment test (FAST), and MinEigen [44]. Among them, the MinEigen [45] method is suitable for compensating the camera movement as in this study, because feature points are extracted for image tracking with camera movement. In addition, feature points are extracted by assuming an affine transformation, as shown in Equation (6), and are evaluated using the degree of dissimilarity as good or bad. Performance comparisons between MinEigen and other methods have been conducted in several studies [44,45,46,47].

Feature points can be matched using various methods, but in this study, random sample consensus (RANSAC) was applied. RANSAC is used to remove outliers and obtain reliable data. It is one of the methods for outlier detection, which is a type of anomaly detection [48]. RANSAC randomly takes some of the data and derives a consensus with the most appropriate fitting model for the data. It classifies inliers and outliers by setting the allowable tolerance. While repeating the random data, the fitting model that maximizes the inlier is selected as the optimal fitting model. In the feature point matching process, an optimal transformation matrix (Equation (6)) is derived through geometrical alignment among the feature points.

When the transformation matrix is obtained from image matching, camera movements, such as pan, tilt, zoom, and rotation, can be inferred. Thus, because the transformation matrix obtained in this process represents the camera movement error, only the pure displacement of the marker can be measured by excluding the camera movement, as shown in Equation (5).

#### 2.1.3. Anomaly Detection

In the process of image matching, an error may occur because feature points are automatically extracted and matched; therefore, the transformation matrix extracted from image matching may contain an error. To correct this error, the displacement is calculated using fixed markers. First, a fixed marker and a moved marker are distinguished through changes in the positional relation among the markers in the images. Then, the transformation matrix is calculated not by image matching, but by using markers with no displacements. In this case, the transformation matrix represents the camera movement error. Finally, the transformation matrix element of the fixed marker is removed from the transformation matrix of the moved marker to measure the pure displacement without camera movement error.

Anomaly detection is used to distinguish between a marker with a displacement and that without a displacement. In this study, outlier detection, which is a type of anomaly detection, is performed based on Chauvenet’s criterion [49]. To apply this method, the structure to be observed must not move as a rigid body, and the marker displacement must occur as an anomaly. Therefore, this method can be applied in construction sites where anomaly displacement occurs, such as earth retaining structures [5,6], steel structures [50], and buildings [51,52].

When all observation data $\hat{x_{i}}\left(i=1,2,\ldots ,n\right)$ follow the normal distribution and have a mean $\bar{x}$ and standard deviation *s*, the Z-score of $\hat{x_{i}}$ is given by Equation (9).

(9) $$z_{i}=\frac{\hat{x_{i}}-\bar{x}}{s}$$

If the probability of occurrence of an observation $\hat{x_{i}}$ is more than $n^{-1}$, according to Chauvenet’s criterion it is regarded as an inlier. In this study, the significance level of Chauvenet’s criterion was set as 0.5. Therefore, if the probability of occurrence is smaller than $\;{\left(2n\right)}^{-1}$, it is judged as an outlier, and an observation $\hat{x_{i}}$ that satisfies Equation (10) is regarded as an inlier.

(10) $$P\left(Z<\left|z_{i}\right|\right)<1-\frac{1}{2n}$$

Anomaly detection refers to the detection of a phenomenon or an object in which an abnormality has occurred in a broad sense. Outlier detection is an anomaly detection method that detects outliers in quantified data [53]. That is, an anomaly can be detected using this method if it is expressed as numerical data, and outliers can be detected from such data. In this study, a motion vector of markers and a marker network in images are considered as numerical data for outlier detection.

The method for extracting outliers through the motion vectors of markers uses the motion vectors in Figure 4b. Since a motion vector $\vec{d_{i}}$ is two-dimensional, it has both size and direction. Because the markers move along the *x*-axis, outliers are extracted using the x component of $\vec{d_{i}}$.

By contrast, the method using network analysis can be applied if the method using motion vectors misses the detection of the moved marker. This method uses the change in distance among the markers in a network. There will be no change in the distance between markers if there is no displacement in the markers; conversely, there will be a change in the distance between markers if there is a displacement in the markers.

When there are *n* markers to be observed, all of the *n* markers are connected with each other; hence, (*n* − 1) different lines connect a particular marker with others. At this time, a change in the length of the segment of the network *n*(*n* − 1)/2 is observed. When $l_{ij}(i\neq j,\;j<j)$ is the initial length of the line segment connecting marker *i*(1,...,(*n* − 1\)) and marker *j*(2,...,*n*), and $l_{ij}^{\prime }(i\neq j,\;j<j)$ is the length of the final segment, the length change $l_{ij}\;$ is expressed as Equation (11).

(11) $$\Delta l_{ij}=\left|{l^{\prime }}_{ij}-l_{ij}\right|$$

The sum $S_{\Delta lk}$ of the change in length of the networks connecting the *k_th_* marker is expressed as Equation (12), and the sum of the initial length $S_{lk}$ of the network connection *k_th_* is given by Equation (13).

(12) $$S_{\Delta lk}=\overset{k-1}{\underset{i=1}{\sum }}\Delta l_{ik}+\overset{n}{\underset{j=k+1}{\sum }}\Delta l_{kj}$$

(13) $$S_{lk}=\overset{k-1}{\underset{i=1}{\sum }}l_{ik}+\overset{n}{\underset{j=k+1}{\sum }}l_{kj}$$

In this experiment, the variance *x_k_* was set as the sum of the length difference of the *k_th_* marker $S_{\Delta lk}$ divided by the sum of the initial length $S_{lk}$ of the *k_th_* marker, as indicated in Equation (14).

(14) $$x_{k}=\frac{S_{\Delta lk}}{S_{lk}}\left(k=1,\ldots ,n\right)$$

In this study, it is assumed that the vector size and the network length change follow a normal distribution. Because the number of markers (*n*) is 12 and the significance level of Chauvenet’s criterion is 0.5, the significance level of anomaly detection is set as ±2.037 based on the Z-score according to Equation (10).

In the case of the model using anomaly detection, the accuracy of detecting a moved marker must be verified before the measurement, because the displacement is measured only for markers that are expected to be moved. For this reason, a test to verify the accuracy of the model in detecting the occurrence of marker displacement using anomaly detection was performed in this study.

### 2.2. Models

**Figure 6:**
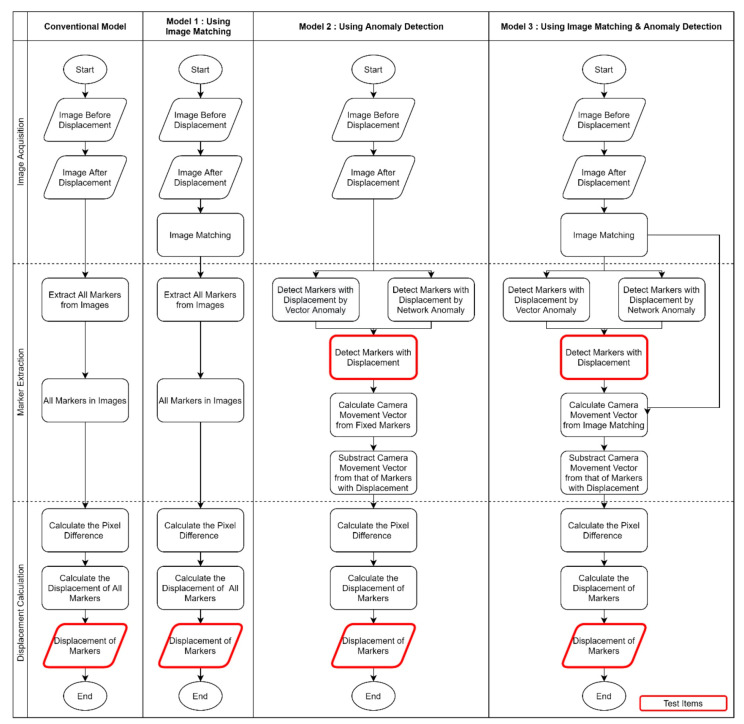
Flowchart of designed models compared with the conventional model.

The methods derived for correcting errors caused by camera movements comprise the following (Figure 6):

1. A method for removing the cause of errors by modifying the step where the errors occur (using image matching, Model 1);
2. An error correction method based on data estimated to be true (using anomaly detection, Model 2);
3. A method for modifying the step and error correction with the data estimated to be true (using both image matching and anomaly detection, Model 3).

#### 2.2.1. Model 1: Using Image Matching

First, to correct errors due to camera movements occurring in the image acquisition step, a model using image matching is presented (Figure 6). In Model 1, the image matching process is performed after the initial and final images are acquired in the image acquisition step. The final image is corrected to fit the initial image using image matching. This will correct the camera movement error by eliminating the differences in the coordinate systems of the images due to camera movements. The position of the marker in the final image has the same image coordinate system and the same camera coordinate system as that of the marker in the initial image. Therefore, the errors of the marker displacement due to camera movements calculated from the initial and final images are corrected.

#### 2.2.2. Model 2: Using Anomaly Detection

The second model is a method for calculating the displacement with only fixed markers using anomaly detection. Anomaly detection is used to distinguish between fixed and moved markers (Figure 6). The anomaly of markers is based on the size of the marker displacement vector in the images and the total length change in the total length of the network segment between each marker (Equation (14)).

In Model 2, anomaly detection is used in the marker extraction step after both the initial and final images are acquired. In this step, the initial and final images are used to classify the markers into those with displacement and those without displacement (Equation (10)). In Equation (7), the pure displacement vector of the marker excluding camera movement is assumed to have no displacement, and the observed displacement vector contains an error. When the error due to camera movement is removed from the observed displacement vector of the marker, the pure displacement vector of the moved marker is derived.

#### 2.2.3. Model 3: Using Both Image Matching and Anomaly Detection

Finally, a method applying both image matching and anomaly detection is proposed (Figure 6). After matching the final image to the initial image using image matching in the image acquisition step, only the markers expected to move are extracted during the marker extraction step. Because both image matching and anomaly detection are used, two corrections are implemented: first with the camera motion vector obtained by image matching and second with the camera motion vector obtained through the fixed marker.

### 2.3. Test Environment

#### 2.3.1. Experimental Setup

**Figure 7:**
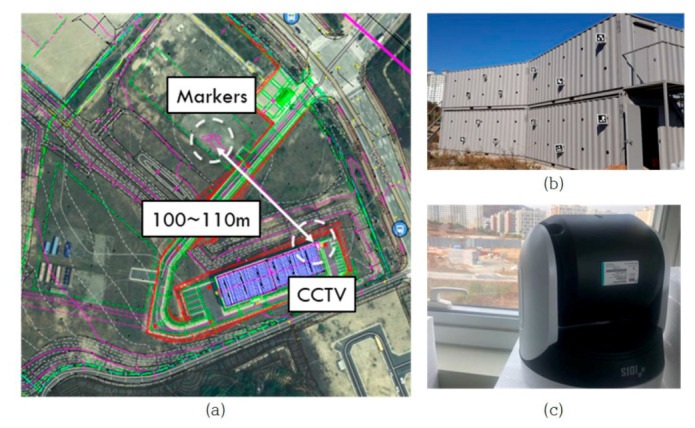
Experimental setup: (**a**) location of markers and closed circuit television (CCTV); (**b**) markers on the container; (**c**) CCTV in the building.

An experimental setup was constructed to analyze the accuracy of the models. The test setup was installed at the POSCO Research Center in Chungju, Chungcheongbuk-do (37.011471°N, 127.829427°E) (Figure 7a). To represent actual structures, four containers were placed, and a marker and a displacement generator were installed (Figure 7b). The displacement generator and 12 markers were installed on the container surface. The size of one container was 6 m long, 3 m wide, and 3 m high, and two buildings were installed on two floors (containers) each. To reduce the error due to environmental factors (except the camera movement error), each container was installed on a concrete foundation, and elements that could generate vibrations were blocked in advance using personnel, vehicles, construction, and other experiment control measures within a 100 m radius from the container.

The camera was installed at horizontal and vertical distances of approximately 100 m and 40 m, respectively, from the container, and the distance from the camera to the markers ranged from 100 to 110 m. It was installed indoors to exclude external environmental factors such as snow, rain, and wind.

**Figure 8:**
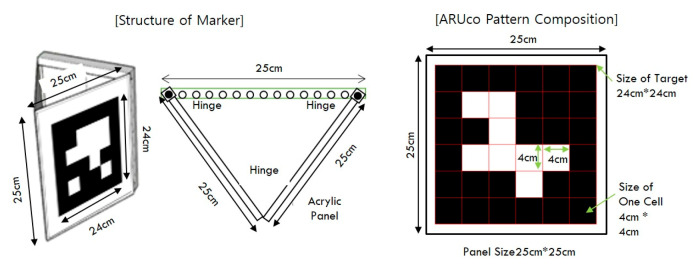
Size of marker and pattern.

The marker arrangement used was the ARUco pattern [40]. One marker consisted of two panels. The size of one panel was 25 cm in width and height, with a 3 mm thick matte acrylic, and it was produced by silk printing with a marker pattern on the panel (Figure 8). There were 12 printed patterns numbered 1 to 11 and number 13. The markers consisted of 36 cells, six rows, and six columns, each 4 cm wide, and were printed according to the ARUco binary block rule. The two panels were connected by hinges with an adjustable angle.

The camera used was IDIS MNC5880SR. It has a resolution of 4K ultra high definition (UHD) (3840 × 2160) and the image sensor is 1/1.7″ CMOS. The image was taken at 10× optical zoom, and the angle of view of the camera at the time of the experiment was approximately 6° horizontally and 3.5° vertically. The size of one marker in the image was approximately 60 pixels to 90 pixels horizontally and vertically, respectively. Table 1 lists the specifications and performance of the installed camera. The camera was calibrated in advance using a full projection matrix with two chessboard markers. One had five columns and six rows, and the size of each cell was 3 cm in width and height. The other had eight columns and 11 rows, and the size of each cell was 2.1 cm in width and height.

#### 2.3.2. System Configuration

For a smooth test environment, a system was installed such that both markers and cameras could be operated remotely. The marker attached to the container was installed with a displacement-generating device. The displacement generator can be operated through the Message Queuing Telemetry Transport (MQTT) protocol and can move the marker forward and backward relative to the face of the container. The video from the camera was transmitted through the real-time streaming protocol (RTSP). The video received through the RTSP was saved as a three-channel jpg file of 3840 × 2160 pixels.

### 2.4. Tests

To determine the camera movement error correction performance and displacement measurement accuracy of the proposed models, the verification objects of the models were set, the experimental items were designed, and the experiment was performed.

#### 2.4.1. Verification Objects

The goal of the experiment was to verify the accuracy of the measured displacement by correcting the camera movement error using each model. The specific performance indices are as follows:

1. Performance of correcting errors due to camera movement using fixed markers;
2. Accuracy of detecting markers with displacement during anomaly detection;
3. Accuracy of measuring displacement.

The performance of correcting errors is used to determine the pure error elimination, except for the systematic error according to the length of displacement. As shown in Equation (7), when the actual displacement of the marker is 0, the systematic error according to the displacement is eliminated; hence, the measured value of the marker without displacement corresponds to the camera movement correction error. Therefore, the performance is verified by measuring the displacement of the fixed marker without displacement.

Because the model using anomaly detection calculates the displacement of a moved marker using the fixed marker, it is important to distinguish between a marker with a displacement and that without one. Therefore, the performance can be verified by false positives and false negatives. A false positive means that the marker is detected as having moved even though it has not moved in reality. By contrast, a false negative indicates that the marker is detected as fixed even though it has moved in reality.

The final verification index is the accuracy of the displacement measurement when camera movements occur. For each model, the measurement accuracy was determined by the movement angle and displacement length. For the displacement, the value measured by the total station is regarded as the true value, and the displacement value obtained using the model is compared to analyze its accuracy and error. Based on these verification indices, the performances of the models are compared and analyzed, and their suitability is evaluated.

#### 2.4.2. Scenarios and Data Acquisition

To verify the performance of each model, the marker displacement and camera movement were artificially generated both in a predetermined order and randomly. The camera movement occurred only during panning (*x*-axis direction, left and right). The horizontal angle of view of the camera was 6°, but because the markers were located at an image range of 4.3°, the maximum horizontal movement of the camera was limited to 1.7°. As the panning movement of the camera could not be accurately controlled, the angle at the camera movement was measured after the image acquisition of each movement. In addition, the displacements of the markers cannot be accurately controlled by the displacement-generating device, and thus these displacements were measured using a total station (Topcon, GM-100) at a distance of 35–40 m from the marker with a measurement error within 2 mm [55]. Because the conventional model has a lot of error because of camera movement, and the error of total station is a type of random error, the displacement measured by the total station is regarded as true displacement without error by repetitive measuring. The data were acquired through six designed scenarios. In total, 79 images (six in the initial state and 73 in the state after displacement or camera movement) were acquired, and 948 sets (72 sets in the initial state and 888 sets in the state after displacement or camera movement) of markers with IDs and positions were obtained from the images (Table 2). The experiment was conducted for an hour from 16:00 to 17:00 on 22 June 2020. At that time, the temperature, humidity, and wind velocity were 34.4 °C, 40%, and 8.3 m/s in a southwesterly direction, respectively.

In Scenarios 1 and 2, images were taken with all fixed markers. Five images were taken in Scenario 1, and the camera pan was sequentially generated to the right four times after acquiring the initial image. The final images were acquired every time a movement occurred.

In Scenario 2, camera movements were randomly generated. Each time, there was a 50% probability that the camera would not move, a 25% probability that the camera would move to the right (+), and a 25% probability that the camera would move to the left (–). After acquiring the initial image, 10 random movements were generated, and the final images were acquired each time. Table 3 presents the camera movement angles for the images acquired from Scenarios 1 and 2.

In Scenarios 3-1, 3-2, and 3-3, both camera movement and marker displacement occurred, and displacement was generated for only one marker per scenario while the others were fixed. The markers and displacements generated in Scenarios 3-1, 3-2, and 3-3 are listed in Table 4, and the direction toward the camera from the container was set as negative (−), while the opposite direction was set as positive (+). The movement of the camera occurred in a specific order within 0–1.7°.

Scenario 4 was conducted with both camera movement and marker displacement, which occurred randomly. The displacement occurred only in marker 7, while the others were fixed. Every time during this scenario, the probability of no displacement occurring at marker 7 was set as 50%, the probability of moving from the container surface toward the camera direction (−) was 25%, and the probability of moving from the container surface to the opposite direction (+) was set as 25%. In the case of the camera, as in Scenario 2, the camera movement had a 50% probability of no movement, a 25% probability of moving to the right (+), and a 25% probability of moving to the left (−). The marker and the camera moved independently, and images were acquired 10 times after the initial image acquisition by randomly generating movements each time. Table 5 presents the randomly generated marker displacement and camera movement angle.

## 3. Results

Using the obtained data, the results of the three experimental indices were derived. The camera movement error correction performance of all models, marker displacement detection performance of Models 2 and 3 using anomaly detection, and displacement measurement accuracy of all models were analyzed.

### 3.1. Correction of Camera Movement Error of Fixed Markers

**Figure 9:**
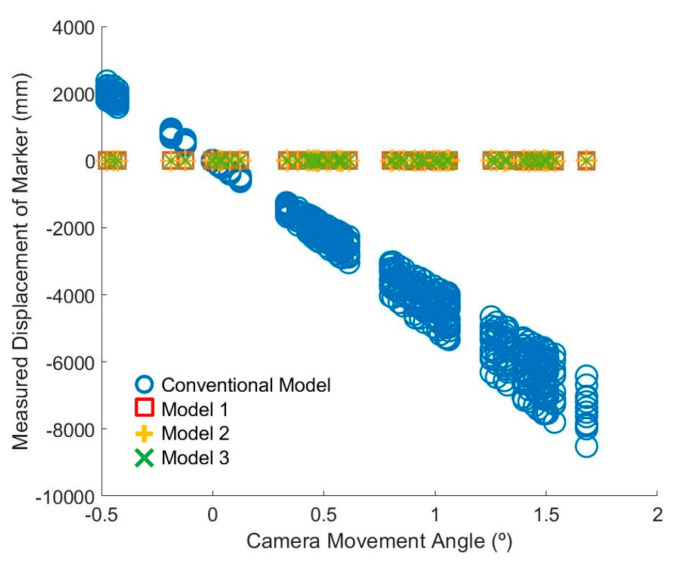
Measured displacement of fixed markers by model vs. camera movement angle.

Through Scenarios 1 and 2, the displacement measurement results of the fixed markers were derived (Figure 9). Because the markers were fixed and only the camera was moved, the displacement measurement results became the camera movement correction residual (Equation (7)). The closer the value to zero, the more accurate the result. Moreover, the smaller the magnitude and standard deviation of the absolute value, the better the performance.

Figure 9 shows the displacements of the fixed markers measured using the conventional model and the proposed models according to the camera movement angle. The results obtained using the conventional method indicate that this method was not able to correct the error due to camera movement: the results have errors of several meters or more depending on the position of the marker (Figure 9). However, the results obtained using the models proposed in this study have errors within several tens of millimeters (Figure 9).

**Figure 10:**
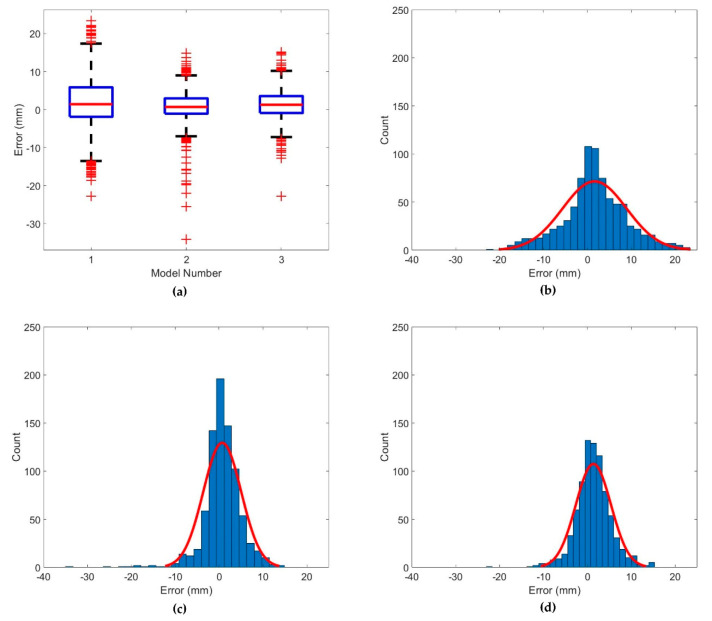
Distribution of errors in the proposed models: (**a**) box plot of error distribution in the models; (**b**) histogram of error distribution in Model 1; (**c**) histogram of error distribution in Model 2; (**d**) histogram of error distribution in Model 3.

Figure 10 depicts the residual distribution of the models, and Table 6 compares the camera error correction performance among models based on the residual distribution. The model with the highest trueness is Model 2 (Figure 10c), and the most precise model appears to be Model 3 (Figure 10d). The largest residual value occurs in Model 2 as the lowest residual (Figure 10c). However, in the case of Models 2 and 3, the displacement of the fixed marker is measured as 0, and the measured displacement is more accurate than the correction property of the camera movement error.

### 3.2. Detection of Marker Displacement

**Figure 11:**
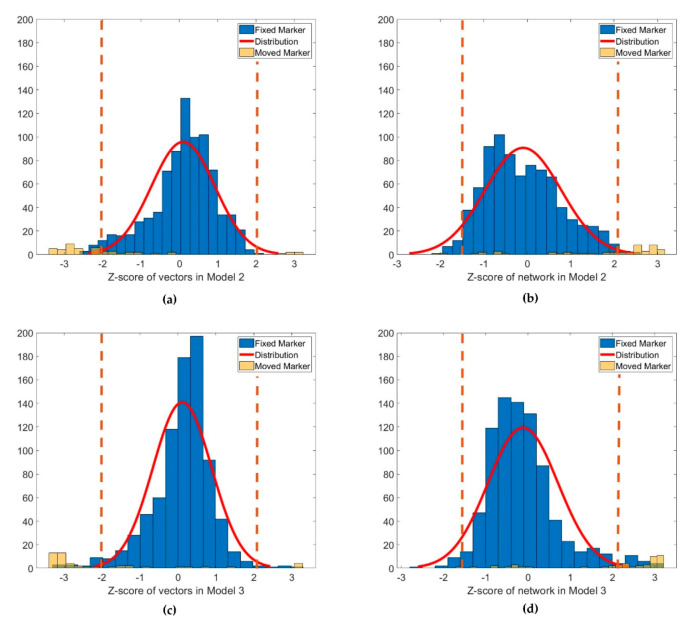
Z-scores of each method in Models 2 and 3: (**a**) Z-score of marker vectors in Model 2; (**b**) Z-score of network length in Model 2; (**c**) Z-score of marker vectors in Model 3; (**d**) Z-score of network length in Model 3.

The performance of the anomaly detection was evaluated. For Models 2 and 3, vector analysis, network analysis, and both were applied. The Z-score distribution of the variation according to each method is displayed in Figure 11. The significance level was set as Z-score = 2.037, according to Chauvenet’s criterion, as given by Equation (10). The total number of datasets was 876, and the number of datasets for markers with displacement was 48. The number of datasets for markers without displacement was 828.

Table 7 lists the specificity and sensitivity of anomaly detection for each method in Models 2 and 3. Each model used three methods for anomaly detection. The first method used only vector analysis, the second only network analysis, and the third used the OR logical operation of Methods 1 and 2. As indicated in Table 7, the largest specificity of 98.8% occurred when only network analysis was used in Model 2 without image matching. On the contrary, the largest sensitivity of 95.8% occurred when only vector analysis was performed in Model 3, where image matching was performed. Two cases where both specificity and sensitivity were measured to be 95% or higher are those using the vector analysis in Model 3.

### 3.3. Displacement Measurement Accuracy

**Figure 12:**
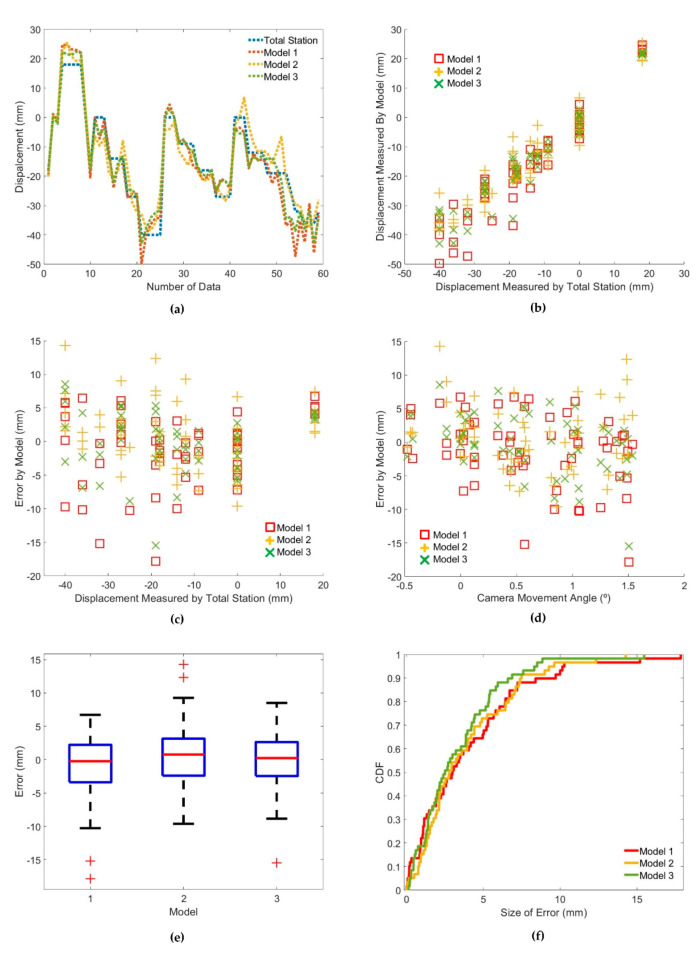
Results of displacement measurement by model: (**a**) measured displacement using total station and the proposed models; (**b**) displacement measured by the models vs. displacement measured by the total station; (**c**) errors of each model vs. displacement measured by the total station; (**d**) errors of each model vs. camera movement angle; (**e**) box plot of error for each model; (**f**) cumulative density function of error size in each model.

The displacements measured by each model and the total station model were compared and analyzed. As illustrated in Figure 12a,b, the displacement measurement accuracy was within several centimeters despite the camera movement. The measurement error of each model did not seem to have a significant correlation with the actual displacement of the markers (Figure 12c). In addition, the correlation between the measurement error of each model and the camera movement angle did not seem to be significant (Figure 12d).

To determine the performance of each model, the error in each model and the size of the error (absolute value of the error) were used. The distribution of errors in the model is depicted in Figure 12e. The distribution of the error size of each model follows the gamma distribution, and the gamma distribution parameters for each model are summarized in Table 8. Despite the camera movement, the size of the error in each model was within 4 mm on average and up to 20 mm. The normalized mean square error (NMSE) was distributed between 0.07 and 0.12. In particular, the probability that the error size of Model 3 would be more than 10 mm was 1.7%, which is less than 5% at a 95% confidence interval (Figure 12f, Table 8). As a result of comparing the trueness and precision of each model based on the distribution of errors, the model using both image matching and anomaly detection showed the highest performance. In particular, in the case of Models 2 and 3, which applied the anomaly detection method, the displacement measurement accuracy was higher than that of the camera movement error correction.

## 4. Discussion

In this study, errors due to camera movements when measuring marker-based structural displacements were corrected using image matching and anomaly detection. The experimental results show that the measurement models applying the image matching and anomaly detection methods are effective in correcting errors. In the experimental environment, the conventional model incurred a maximum error of 8000 mm or more; however, by using the models proposed in this study, the error due to camera movement was reduced to within 30 mm. The error can be reduced to less than 20 mm by excluding the fixed markers through anomaly detection and measuring only the moved markers. It was confirmed that this error was not correlated with the camera movement angle and the amount of marker displacement.

In particular, in the case of Model 3, which used both image matching and anomaly detection, the moved markers were detected with a specificity of 98.7% and a sensitivity of 95.8%. The average and maximum measurement errors were approximately 3.29 and 15.47 mm, respectively, and the NMSE of this model was 0.074. The probability that the error size would be more than 10 mm was 1.7%, which is less than 5% at a 95% confidence interval.

The models presented in this study are suitable for measuring the distance of structures. For example, in an earth-retaining wall, local displacement of at least 15 mm begins one month before the collapse [5,6], and deformation of the high rock slopes during highway construction would occur within 15 mm if stable [10]. In addition, displacement on the roof of a building shows signs of deformation when it is more than 0.03 times the height of the building, and the roof tends to collapse when displacement is larger than 0.07 times the height of the building [52]. This means a displacement of 300 mm and 700 mm for a 10 m building. Given the distribution of errors in the models, it can be determined that this model is suitable for displacement measurement of such structures.

Despite the above performances, this study has the following limitations. The models proposed in this study can only correct errors due to panning and tilting of the camera, but other errors cannot be corrected. Therefore, a follow-up study is required to correct such errors, e.g., those caused by other movements, such as camera rotation and zoom. In addition, if the structure to be measured undergoes rigid motion or if there is no anomaly in the displacement of the structure, the camera movement and the displacement of the structure are indistinguishable because the part of the structure that is without movement is used to compensate for the error. Therefore, to apply the studied models, the tendency of displacement of the structure must be known in advance. Finally, the measurement of the marker displacement was limited to a two-dimensional plane; thus, additional cameras are necessary for three-dimensional displacement measurement, or additional studies are necessary for a more accurate three-dimensional marker-based displacement measurement with only one camera.

Nevertheless, this study is significant in the following aspects. First, the camera movement was corrected over long distances of ≥100 m. Previous studies were conducted at short distances (less than 50 m), with completely fixed cameras to exclude camera movement, or with an accuracy of several centimeters or more. However, the models developed in this study can be applied at an actual construction site, where camera movements exist or structural displacement measurement from a long distance is required. Second, the cost of camera movement error correction is low when the developed models are used. In the conventional method, to compensate for camera movements, an IMU sensor or an additional device are used. If low-cost IMU sensors are employed, measurement errors occur [56], making it difficult to correct camera movements. Therefore, it is necessary to use an expensive IMU sensor [39] or an additional device to fix the camera. However, in the proposed method, an additional device is not required because the measurement results are used to correct errors through a mathematical model. Moreover, the need for an expensive camera is avoided, as only a low-cost camera, i.e., that of a common CCTV, is required.

## 5. Conclusions

In this study, models were developed to correct camera movement errors using image matching and anomaly detection, and their performances were verified by comparing them with that of the conventional model (total station). Although there were some differences in the performance of each model, the detection rate of markers with displacement reached 95%, and the probability of the error size to be less than 10 mm was ≥95% with a 95% confidence interval when both image matching and anomaly detection were used. Therefore, even with a low-cost 4K camera over a long distance of 100 m or more, an accurate marker-based displacement measurement can be achieved. In particular, model 3 was found to be the best suited to compensate for camera movement error.

In future research, we intend to overcome the limitations of the models developed in this study and increase their usability. To overcome the limitations of these models in which markers must be attached, we will study a method for automatically extracting feature points from the image and extracting the structural displacement based on the feature points. In addition, by changing the range of camera motion in various ways, we intend to develop a displacement measurement model that can be applied to mobile image acquisition devices, such as unmanned aerial vehicles, mobile phones, and mobile mapping systems, without limiting the error correction range to camera movements. Furthermore, we plan to achieve a higher displacement measurement accuracy compared to those of the models in this study.

## Figures and Tables

**Table 1:** Specification of CCTV [54].

Item	Specification
Image sensor	1/1.7″ Complementary metal-oxide-semiconductor (CMOS)
Maximum resolution	3840 × 2160
Scanning method	Progressive scan
Lens type	Autofocus (AF) zoom lens
Focal length	f = 6.5–202 mm (31×)
View angle	Wide: 58.2° (H), 34.4° (V), 65.2° (D) Tele: 1.99° (H), 1.13° (V), 2.3° (D)

**Table 2:** Number of datasets.

Scenario Number	Camera Movement	Marker Displacement	Marker ID with Displacement	Number of Datasets	
				Images	Markers
1	In order	None	None	5 (1 + 4)	60 (12 + 48)
2	Random	None	None	11 (1 + 10)	132 (12 + 120)
3-1	In order	In order	2	16 (1 + 15)	192 (12 + 180)
3-2			7	16 (1 + 15)	192 (12 + 180)
3-3			13	20 (1 + 19)	240 (12 + 228)
4	Random	Random	7	11 (1 + 10)	132 (12 + 120)
			Total	79 (6 + 73)	948 (72 + 876)

**Table 3:** Camera movement angles (°) in Scenario 1 and Scenario 2.

Scenario Number	0	1	2	3	4	5	6	7	8	9	10
Scenario 1	0.000	0.428	0.908	1.400	1.681	-	-	-	-	-	-
Scenario 2	0.000	0.000	0.472	0.472	0.912	0.469	0.468	0.468	0.469	0.468	0.469

**Table 4:** Marker ID with displacement and corresponding displacement values in Scenarios 3-1, 3-2, and 3-3.

Scenario Number	Marker ID with Displacement	Marker Displacement (mm)
3-1	2	−14, −27, −40
3-2	7	−9, −18, −27
3-3	13	−12, −19, −25, −32, −36

**Table 5:** Marker displacement and camera movement angle in Scenario 4.

Scenario Number	0	1	2	3	4	5	6	7	8	9	10
Displacement (mm)	0	−18	0	18	18	18	18	18	18	0	−18
Camera Angle (°)	0.000	−0.001	0.000	−0.475	−0.002	0.479	0.042	−0.445	−0.447	0.029	−0.427

**Table 6:** Residual by model after correcting the camera movement error (mm).

	Mean	Standard Deviation	Lower Maximum	Upper Maximum
Model 1	1.63	7.28	−22.78	23.37
Model 2	0.71	4.33	−34.12	14.83
Model 3	1.36	4.00	−22.78	15.14

**Table 7:** Anomaly detection performance by method.

Model	Anomaly Detection Method	False Positive ^1)^	False Negative ^2)^	Specificity ^3)^	Sensitivity ^4)^
Model 2	Vector	14	14	98.3%	70.8%
	Network	10	18	98.8%	62.5%
	Vector or Network	23	12	97.2%	75.0%
Model 3	Vector	11	2	98.7%	95.8%
	Network	18	8	97.8%	83.3%
	Vector or Network	21	2	97.5%	95.8%

^1)^ Detected as moved but fixed in reality; ^2)^ detected as fixed but moved in reality; ^3)^ true negative/(true negative + false positive); ^4)^ true positive/(true positive + false negative).

**Table 8:** Error distribution of models.

Model	$E{\left(\left|e\right|\right)}^{\;1)}$ **(mm)**	$Max{\left(\left|e\right|\right)}^{\;2)}$ **(mm)**	NMSE ^3)^	Γ-Distribution		**Confidence Interval of** $p{\left(\left|e\right|>10\;\mathrm{mm}\right)}^{\;4)}$		
				α	β	Lower Limit	Mean	Upper Limit
1	3.952	17.849	0.116	0.927	4.264	0.004	0.068	0.132
2	3.806	14.270	0.095	1.308	2.910	0.000	0.034	0.080
3	3.290	15.469	0.074	1.331	2.781	0.000	0.017	0.050

^1)^ Average size (absolute value) of error; ^2)^ maximum size of error; ^3)^ Normalized mean square error; ^4)^ probability that the size of error is more than 10 mm.
